# The Categorical Distinction Between Targets and Distractors Facilitates Tracking in Multiple Identity Tracking Task

**DOI:** 10.3389/fpsyg.2016.00589

**Published:** 2016-04-28

**Authors:** Liuqing Wei, Xuemin Zhang, Chuang Lyu, Zhen Li

**Affiliations:** ^1^Beijing Key Lab of Applied Experimental Psychology, School of Psychology, Beijing Normal UniversityBeijing, China; ^2^State Key Lab of Cognitive Neuroscience and Learning and IDG/McGovern Institute for Brain Research, Beijing Normal UniversityBeijing, China; ^3^Center for Collaboration and Innovation in Brain and Learning Sciences, Beijing Normal UniversityBeijing, China; ^4^eMetric, LLC, San AntonioTX, USA

**Keywords:** Multiple Identity Tracking, semantic category, category-based grouping effect, inter-category distinction, visual distinctiveness

## Abstract

This study investigates the tracking facilitation effect during categorical distinction between targets and distractors in the Multiple Identity Tracking task. We asked observers to track four targets in a total of eight moving objects, and manipulated categorical distinctions of targets and distractors across four experiments, with different combinations of inter-category and intra-category differences. Results show that tracking performance was significantly better when the targets and distractors were inter-category different, compared to when the targets and distractors were identical or intra-category distinctive. As the inter-category distinction between targets and distractors narrowed, tracking performance improved, but the inter-category facilitation effect decreased. These results may indicate a category-based grouping effect: the observers organized the targets within the same semantic category into one group and made the targets more easily and accurately rediscovered when lost during tracking. Furthermore, the tracking facilitation of categorical distinction diminished when all the objects were inverted. This proved that besides their visual distinctiveness, objects’ semantic category information also played an important role during tracking.

## Introduction

Multiple Object Tracking (MOT) tasks have been used to study the ability of humans to keep track of multiple moving objects and the cognitive processing of visual attention (Pylyshyn and Storm, 1988; Scholl, 2009). In the traditional MOT tasks, multiple identical objects are presented with a subset designated as “targets”. Observers are asked to track the randomly moving targets for several seconds. The observers’ ability to identify the targets reflects their ability to maintain object representation over time and space. Different from MOT, Multiple Identity Tracking (MIT) tasks employ objects with unique features (such as different colors, numbers, animals, or human faces) as tracking stimuli to investigate the effects of unique object features or identities on tracking performance and identity processing in dynamic scenes (Oksama and Hyönä, 2004, 2008; Makovski and Jiang, 2009a,b). In the real world, people process not only the surface features of the objects, but also their semantic meanings. Without much conscious effort, people are able to recognize the objects in the environment and organize them into different categories (Caramazza, 1998; Shelton and Caramazza, 2000). In this study, we explore the effect of categorical organization on dynamic object tracking, by applying objects carrying categorical information in a variant of MIT task.

Previous researches have examined the effects of visual distinctiveness or the uniqueness of objects’ features on tracking performance. Makovski and Jiang (2009a,b) found that participants’ tracking performance improved when the targets differed from the distractors in single feature such as color, shape, or digit. For example, in the all-unique condition, the eight objects had eight different colors. The tracking performance of this condition improves upon the homogenous condition where the eight objects were identical in color. However, the improvement of tracking performance disappeared when the targets shared the features of the distractors but differed in the combination of target-distractor features (Makovski and Jiang, 2009b). In their study, for example, in the conjunction-distinct condition, eight distinct objects produced by the conjunction of four colors and four digits. No two objects had the same combination of color and digit, but a given target (e.g., a red 5) shared color with one of the distractors (e.g., a red 4) and digit identity with another distractor (e.g., a green 5). Compared to the homogenous condition, tracking performance was disturbed in this condition. This could be explained by that the feature binding during attentional tracking was restricted to a limited degree, which resulted in the uniqueness of features’ conjunction did not improve tracking performance (Makovski and Jiang, 2009b). Howe and Holcombe (2012) argued that conjunction targets could improve tracking performance in some conditions. In their study, for example, when the targets were small red squares, half the distractors were large red squares and the other half were small green squares, tracking performance increased relative to the homogenous feature-conjunction condition. The condition in which the targets were green squares with red centers and the distractors were red squares with green centers also enhanced tracking. In these conditions, the target set was guidable and directed more attention to the targets than distractors. Another possible explanation was that observers might group the targets together based on their feature distinctiveness and segregate them from the distractors. Feria (2012) provided evidence that additional distractors that differ from the targets by one feature (shape, color, or motion) or two features impaired tracking less than distractors that were identical to the targets. Therefore, observers could use the objects’ distinctive features to distinguish targets from distractors during tracking.

Furthermore, Liu et al. (2012) showed that object uniqueness could produce both costs and benefits to tracking performance, mainly depending on the objects’ visual complexity. When object stimuli were less complex, such as numbers of 1 digit and 2 digits length or simple Chinese characters, the unique identity enhanced tracking performance. When the target stimuli were visually complex, such as numbers of 3 digits and 4 digits length or complex Chinese characters, the targets’ uniqueness impaired tracking performance compared to the homogenous condition. The determinants of the facilitation or impairment effect might be the levels of resources taken by identity processing and the limit of working memory capacity. When the target identity was simple and easy to process, unique targets with their surface features stored in working memory could aid targets recovery. When the target identity was complex and had a lower working memory span, unique targets consumed extra cognitive resources and impaired tracking performance. Ren et al. (2009) have reported similar results using multiple-face tracking task, and they found unique upright faces impaired tracking performance relative to identical upright or unique inverted faces. This may be due to that processing of human faces is difficult and requires additional resources.

Besides simple surface features (e.g., color, shape, size, or their combination), Some studies used cartoon animals as tracking objects to investigate the role of object identity during tracking. These studies found that unique objects make tracking easier compared to the homogenous condition and the paired condition (the targets animals also used as the distractors) (Horowitz et al., 2007; Drew et al., 2013).

About the reason why simple unique objects could promote tracking, previous study have suggested that because the unique identities of objects are stored in visual working memory, once one or more targets were lost during tracking, observers could utilize the stored identities to recover the targets (Makovski and Jiang, 2009a,b; Ren et al., 2009; Liu et al., 2012). However, there is another possible mechanism: observers may use the unique identities of objects to discriminate the targets and distractors much clearly. That is, observers grouped the targets together based on their feature distinction (Feria, 2012; Howe and Holcombe, 2012). In the traditional MOT tasks, the grouping hypothesis proposed by Yantis (1992) pointed out: to complete the tracking task, observers initially construct a perceptual representation of a virtual polygon at the targets designation phase, and they continuously update their internal representation of the target configuration along with the motion of the targets. The perceptual grouping processes the targets as a whole virtual object in visual system. Later studies provided more evidence that observers could form a group representation based on spatiotemporal information (for example, the change of motion trajectory, Ogawa and Yagi, 2002; common motion, Suganuma and Yokosawa, 2006). Some researchers focused on the automatic feature-based grouping effect while using color, size, shape, or their combination as objects’ identities (Keane et al., 2011; Erlikhman et al., 2013). These studies found that the surface features automatically bound targets with distractors in one group and impaired tracking performance, when half targets and half distractors possessed one feature token and the remaining objects had another feature token. Feature-based grouping occurred even when it was irrelevant to the task instructions and contrary to the task demands, suggesting that the grouping effect during tracking was automatic to some extent (Keane et al., 2011; Erlikhman et al., 2013).

As we know, visual object knowledge can be organized into different categories innately in reality (Mahon and Caramazza, 2011). These semantic categories of knowledge, such as animals and tools, can be considered to be fundamentally different in contrast to surface feature grouping. Then, questions of interest follow. When the targets and distractors belong to different categories, would observers organize targets into one group during tracking and improve tracking performance? When the categorical distinction between the targets and distractors narrows, would the category-based grouping effect be weakened? To answer these questions, the present study aimed to explore the facilitation effect when targets and distractors belong to different categories, as well as the persistence of the effect as the categorical distinction narrows. Meanwhile, we also intended to examine the effect of objects’ uniqueness within the same category on tracking tasks. It is assumed that the tracking improvement would be less for the objects’ uniqueness within the same category condition, compared with the categorical distinction condition.

We hypothesized that categorical distinction between targets and distractors can be utilized to organize the targets in one group and segregate them from the distractors. The semantic categories may be processed automatically during the target designation phase, and continue to exert an influence over tracking. In the current study, we designed four experiments to investigate the tracking facilitation effect of categorical distinction between targets and distractors. We used typical and nameable objects in this study. These objects are common in the real world, and thus have high ecological validity. In Experiment 1, we used land mammals and furniture as natural and artificial categories, respectively, to test the category-based tracking facilitation effect. We narrowed the categorical difference to land mammals and fruits which are animals and plants in Experiment 2, and further narrowed the categorical difference to land mammals and birds which are both animals in Experiment 3, to examine whether the category-based tracking facilitation effect persisted when there was a smaller categorical difference between targets and distractors. Since we used pictures of line drawings as tracking stimuli, the visual distinctiveness between targets and distractors belonging to different categories was much more evident than that of the same category. In Experiment 4, we inverted pictures and compared the tracking facilitation effects of upright and inverted objects, in order to provide further evidence that the tracking facilitation effect was not only caused by the visual or perceptual distinctiveness between targets and distractors, but also caused by their categorical distinction.

## Experiment 1

In Experiment 1, we used land mammals and furniture as natural and artificial categories to test the category-based tracking facilitation effect and the influence of target uniqueness on tracking performance. The neuropsychological research proposes that semantic knowledge is represented categorically in the brain, and knowledge related to different semantic categories is anatomically segregated accordingly (Caramazza and Mahon, 2003, 2006; Mahon and Caramazza, 2011). From both the empiricist and rationalist perspectives in cognitive psychology, people can generally classify objects as natural, or artificial items (Caramazza, 1998; Caramazza and Shelton, 1998). We assumed that when the targets and distractors belong to different semantic categories, observers would use this distinction to aid tracking, thereby presenting a category-based facilitation effect.

### Methods

#### Participants

Eleven undergraduate students (four female) aged 21–26 years (mean age = 23.27 years, *SD* = 1.56) with normal or corrected-to-normal vision completed the experiment. All observers provided informed written consent. The study was approved by the Institutional Review Board (Ethics Committee) of the School of Psychology at Beijing Normal University. All observers received payment.

#### Equipment and Stimuli

##### Equipment

The experiment task was controlled by the C# programing language. Stimuli were displayed on a Founder 17′ CRT monitor with a resolution of 1024 × 768 pixels and a refreshing rate of 85 Hz. Observers responded by pressing a keyboard and a mouse.

##### Stimuli

The moving objects were presented in a white square display that subtended 1024 × 768 pixels (40.96° × 30.72°). There was a central gray fixation cross that subtended 40 × 40 pixels (1.6° × 1.6°). Sixteen pictures were selected from Cycowicz et al.’s (1997) set of 400 line drawings. The eight land mammals’ pictures were of a bear, cat, dog, donkey, horse, lion, rabbit, and lamb. The eight furniture pictures were of a bed, chair, couch, desk, dresser, rocking chair, stool, and bench. Each picture was fit to a square of 60 × 60 pixels (2.4° × 2.4°) with an imagery boundary.

The initial locations of the pictures, moving at a horizontal and vertical velocity between -5 and +5 pixels per frame at random, were randomly assigned (Pylyshyn, 2006; Pylyshyn et al., 2008). Each object changed its speed and direction randomly at each frame. A repulsion technique was adopted to keep the circles from colliding. The objects bounced off each other when the center-to-center distance was less than 60 pixels. They also avoided the edge of the display area. The maximum speed for an object was 24.1°/s, and the minimum speed was 3.4°/s.

#### Design

The experiment was a single-factor within-subject design. The independent variable was the semantic category distinction of targets and distractors with eight conditions. In the intra-category conditions, the targets and distractors were selected from the same category. In the inter-category conditions, the targets were selected from one category, while the distractors were selected from another category. The homogenous condition provided the performance baseline for comparison with the other levels (see **Table 1**). In the homogenous level, all of the targets and distractors were the same and were randomly chosen from either the set of land mammals or furniture. **Table 1** presents the details of our experiment design.

**Table 1:** The eight conditions of Experiment 1.

Conditions	Levels	Targets	Distractors
Intra-category	Homogenous	Land mammals A, land mammals A, land mammals A, land mammals A	Land mammals B, land mammals B, land mammals B, land mammals B
	Four-unique	Land mammals A, land mammals A, land mammals B, land mammals B	Land mammals C, land mammals C, land mammals D, land mammals D
	All-unique	Land mammals A, land mammals B, land mammals C, land mammals D	Land mammals E, land mammals F, land mammals G, land mammals H
	Paired-two	Land mammals A, land mammals A, land mammals B, land mammals B	Land mammals A, land mammals A, land mammals B, land mammals B
Inter-category	Homogenous	Land mammals A, land mammals A, land mammals A, land mammals A	Furniture A, furniture A, furniture A, furniture A
	Four-unique	Land mammals A, land mammals A, land mammals B, land mammals B	Furniture A, furniture A, furniture B, furniture B
	All-unique	Land mammals A, land mammals B, land mammals C, land mammals D	Furniture A, furniture B, furniture C, furniture D
Homogenous		Land mammals A, land mammals A, land mammals A, land mammals A	Land mammals A, land mammals A, land mammals A, land mammals A


*The A, B…H represented the eight land mammals or furniture images. The land mammals and furniture targets and distractors were randomly assigned, although here only one particular combination is shown. Please see the text for more details.*

As shown in **Table 1**, the intra-category conditions included four sub-conditions. In the intra-category homogenous condition, the targets consisted four same land mammals or furniture, while the distractors were another four same land mammals or furniture correspondingly in the same category. Both the targets and distractors were randomly chosen from the sets of eight possible land mammals/furniture images, with the constraint that they could not be the same. The intra-category four-unique condition was similar to the intra-category homogenous condition except that the targets consisted two land mammals or furniture (i.e., half the targets were the same land mammals or furniture and the remaining targets were another different land mammals or furniture) and the distractors were another two in the same category. In the intra-category all-unique condition, eight unique objects were chosen from the same category. In the intra-category paired-two condition, the targets and distractors shared the same identities. They were both two land mammals/furniture (i.e., half the targets/distractors were the same land mammals/furniture and the remaining targets/distractors were another different land mammals/furniture).

The inter-category conditions included three sub-conditions. In the inter-category homogenous condition, the targets were the same land mammals/furniture and the distractors were another different furniture/land mammals, with the constraint that the targets and distractors were chosen from different categories. For example, the targets could be one land mammal’s image chosen randomly from the set of eight possible land mammals and the distractors could be one furniture image chosen randomly from the set of possible furniture images. The inter-category four-unique condition was similar to the inter-category homogenous condition except that the targets contained two land mammals/furniture and the distractors were two furniture/land mammals in a different category. For example, half the targets were rabbits and the remaining targets were lambs, while half the distractors were couches and the remaining distractors were desks. In the inter-category all-unique condition, the targets were four unique land mammals/furniture images and the distractors were four unique furniture/land mammals images.

The dependent variable was tracking accuracy defined as the average proportion of correctly identified targets. As 4 of the 8 objects were targets, chance accuracy was 50%.

### Procedures

Observers sat approximately 57 cm away from the monitors so that each pixel subtended 0.04° of the visual angle. The observers were shown instructions for the tracking task on the screen. At the start of each trial, a gray fixation cross and eight pictures were displayed. The observers were encouraged to maintain their fixation on the central cross during tracking. Four of eight pictures flashed five times in 1 s for observers to identify them as the targets and distinguish them from the distractors. Then, all of the pictures began to move randomly, and the movement stopped at a random point within 5–8 s to avoid observers simply remembering the last frames of the motion instead of tracking the targets. At the end of motion, the pictures were masked by gray squares subtended 60 × 60 pixels (2.4° × 2.4°). Observers were given 20 s to select all four targets with the mouse. Selected squares were highlighted by red frames. Observers pressed the space bar to continue to the next trial. Unlike the traditional MIT task, observers of the present study were not required to bind the targets’ identities and locations together. At the end of the tracking, observers only need to report the locations of the targets. Therefore, observers could achieve tracking entirely by updating the motion trajectories of the targets without remembering their identities.

The experiment began with eight practice trials, one for each condition. The experiment consisted of 160 trials (20 trials × 8 levels of semantic category distinction) displayed in random order. Observers rested for at least 1 min every 40 trials.

### Results and Discussion

**FIGURE 1:**
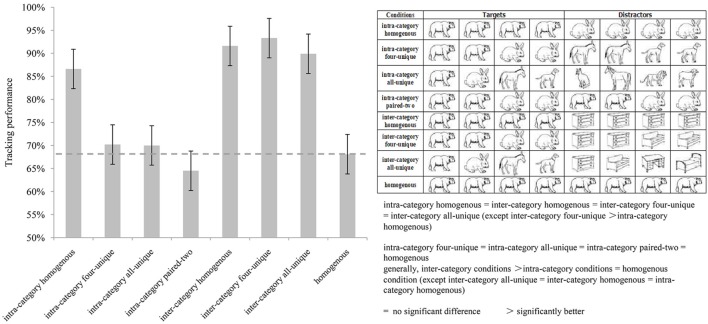
**The results of Experiment 1.** The **(left)** histogram describes the average tracking performance of each condition. The **(right)** above table illustrates the targets and distractors’ identities of each condition. The **(right)** below is the brief results of pairwise comparisons.

The results are shown in **Figure 1**. A repeated measures analysis of variance (ANOVA) indicated a significant main effect [*F*(7,70) = 51.108, *p* < 0.001, η^2^ = 0.836]. *Post hoc* tests with Bonferroni correction showed that there was generally no significant difference (*ps* > 0.05) among the paired intra-category homogenous, the inter-category homogenous, the inter-category four-unique and the inter-category all-unique conditions. One exception is that the tracking performance of the inter-category four-unique condition was significantly better than that of the intra-category homogenous condition, That is, the inter-category difference of the targets and distractors, rather than the identity uniqueness of the targets, played a relatively more important role in improving observers’ tracking performance. However, when the targets and distractors belonged to the same category, the tracking performance was enhanced only when greater identity difference between the targets and distractors existed, such as in the intra-category homogenous condition where the targets were four same objects and the distractors were another four same objects.

In addition, no significant difference was found when we compared tracking performances of the intra-category four-unique, intra-category all-unique, intra-category paired-two, and homogenous conditions in pairs (*ps* > 0.05). This result was different from a previous finding that unique objects could aid tracking in MIT tasks (Horowitz et al., 2007; Drew et al., 2013). The reason might be that the objects used in our experiment consisted of simple line drawings, and their visual angles were relatively small (2.4° × 2.4°). Moreover, the objects’ speed of motion was high (The maximum speed for an object was 24.1°/s, and the minimum speed was 3.4°/s). These parameters might reduce the effect of identities information on tracking performance. On the contrary, the pairwise comparison of tracking performances among all the other conditions showed significantly difference, all *ps* < 0.05 (see **Table 2**).

**Table 2:** The pairwise comparisons of conditions in Experiment 1.

	Intra-category homogenous	Intra-category four-unique	Intra-category all-unique	Intra-category paired-two	Inter-category homogenous	Inter-category four-unique	Inter-category all-unique
Intra-category homogenous							
Intra-category four-unique	0.164ˆ*						
Intra-category all-unique	0.166ˆ**	0.002					
Intra-category paired-two	0.220ˆ***	0.057	0.055				
Inter-category homogenous	-0.050	-0.214ˆ**	-0.216ˆ***	-0.270^∗∗∗^			
Inter-category four-unique	-0.067ˆ*	-0.231ˆ***	-0.233ˆ***	-0.288^∗∗∗^	-0.017		
inter-category all-unique	-0.033	-0.197ˆ***	-0.199ˆ**	-0.253^∗∗∗^	0.017	0.034	
Homogenous	0.185ˆ**	0.022	0.019	-0.035	0.235^∗∗∗^	0.252^∗∗∗^	0.218^∗∗∗^


*The values in the table are the *mean differences* of tracking performances between two conditions. ^∗^*p* < 0.05, ^∗∗^*p* < 0.01, ^∗∗∗^*p* < 0.001.*

Tracking performances of the inter-category conditions were significantly better than that of the intra-category conditions (except for one pair: the inter-category all-unique and intra-category homogenous conditions were not significantly different) and the homogenous condition. This provided evidence that categorical distinction between targets and distractors significantly improved tracking performance. By grouping the targets into one virtual representation, observers might find the targets more easily. These findings are further discussed in the general discussion section.

## Experiment 2

In Experiment 2, to further test the category-based tracking facilitation effect in MIT tasks, we decreased the categorical distinction between the targets and distractors to land mammals and fruits. Though land mammals and fruits are all natural objects, they have many different features, such as shape, status of static or moving, and the relationship with humans. We predicted that the category-based tracking facilitation effect in Experiment 2 decreased from Experiment 1, but still could be detected.

### Participants

Eleven undergraduate students (eight female) who did not participate in Experiment 1 aged 18–26 years (mean age = 23.00 years, *SD* = 2.72) with normal or corrected-to-normal vision completed the experiment. All observers provided informed written consent. The study was approved by the Institutional Review Board (Ethics Committee) of the School of Psychology at Beijing Normal University. All observers received payment for their time.

### Design, Equipment, Stimuli, and Procedures

The materials adopted in Experiment 2 were eight fruits and eight land mammals, which all belong to natural categories. The eight land mammals were the same as those used in Experiment 1. The eight fruit pictures were of an apple, banana, grapes, lemon, peach, pear, pineapple, and strawberry, selected from Cycowicz et al.’s (1997) set of 400 line drawings. The design, equipment and procedures were the same as Experiment 1(see **Table 3**).

**Table 3:** The eight conditions of Experiment 2.

Conditions	Levels	Targets	Distractors
Intra-category	Homogenous	Land mammals A, land mammals A, land mammals A, land mammals A	Land mammals B, land mammals B, land mammals B, land mammals B
	Four-unique	Land mammals A, land mammals A, land mammals B, land mammals B	Land mammals C, land mammals C, land mammals D, land mammals D
	All-unique	Land mammals A, land mammals B, land mammals C, land mammals D	Land mammals E, land mammals F, land mammals G, land mammals H
	Paired-two	Land mammals A, land mammals A, land mammals B, land mammals B	Land mammals A, land mammals A, land mammals B, land mammals B
Inter-category	Homogenous	Land mammals A, land mammals A, land mammals A, land mammals A	Fruit A, fruit A, fruit A, fruit A
	Four-unique	Land mammals A, land mammals A, land mammals B, land mammals B	Fruit A, fruit A, fruit B, fruit B
	All-unique	Land mammals A, land mammals B, land mammals C, land mammals D	Fruit A, fruit B, fruit C, fruit D
Homogenous		Land mammals A, land mammals A, land mammals A, land mammals A	Land mammals A, land mammals A, land mammals A, land mammals A


*The land mammals and fruits targets and distractors were randomly assigned, although here only one particular combination is shown. Please see the text for more details.*

### Results and Discussion

**FIGURE 2:**
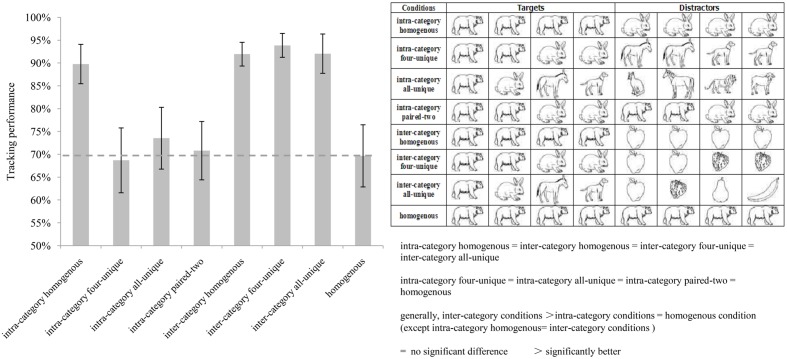
**The results of Experiment 2.** The **(left)** histogram describes the average tracking performance of each condition. The **(right)** above table illustrates the targets and distractors’ identities of each condition. The **(right)** below is the brief results of pairwise comparisons.

**Figure 2** presents the results for Experiment 2. A repeated measures ANOVA indicated a significant main effect [*F*(7,70) = 41.303, *p* < 0.001, η^2^ = 0.805]. *Post hoc* tests with Bonferroni correction (see **Table 4**) showed that most of the pairwise comparisons were significant except for the intra-category homogenous, inter-category homogenous, inter-category four-unique, and inter-category all-unique conditions, for which no significant difference was found between each pair, *ps* > 0.05. This might have the same reason as that in Experiment 1. The objects used in our experiment were simple line drawings with small visual angles and high motion speeds, resulted hard for observers to use targets’ distinct identities to promote tracking. Observers mainly used the categorical distinction between the targets and distractors to aid tracking. Similar to the results of Experiment 1, no significant differences (*ps* > 0.05) were found between each paired of the intra-category four-unique, intra-category all-unique, intra-category paired-two and homogenous conditions.

**Table 4:** The pairwise comparisons of conditions in Experiment 2.

	Intra-category homogenous	Intra-category four-unique	Intra-category all-unique	Intra-category paired-two	Inter-category homogenous	Inter-category four-unique	Inter-category all-unique
Intra-category homogenous							
Intra-category four-unique	0.211ˆ***						
Intra-category all-unique	0.162ˆ**	-0.049					
Intra-category paired-two	0.190ˆ***	-0.021	0.027				
Inter-category homogenous	-0.022	-0.233ˆ**	-0.184ˆ*	-0.211ˆ**			
Inter-category four-unique	-0.041	-0.252ˆ**	-0.203ˆ**	-0.231ˆ***	-0.019		
Inter-category all-unique	-0.023	-0.234ˆ***	-0.185ˆ**	-0.213ˆ**	-0.001	0.018	
Homogenous	0.201ˆ**	-0.010	0.039	0.011	0.223^∗∗^	0.242^∗∗∗^	0.224^∗∗^


*The values in the table are the *mean differences* of tracking performances between two conditions. ^∗^*p* < 0.05, ^∗∗^*p* < 0.01, ^∗∗∗^*p* < 0.001.*

The results also show that the tracking performances of observers in inter-category conditions were significantly better than those in the intra-category conditions and the homogenous condition. One exception is that no significant difference was found between the intra-category homogenous condition and inter-category conditions. These results suggest that the categorical distinction of targets and distractors also improved tracking performance when the categorical difference was between land mammals and fruits, which are all natural objects. A category-based grouping effect might also exist during tracking in Experiment 2.

## Experiment 3

Experiment 3 examined whether semantic category distinction would improve tracking performance when the categorical differences were further decreased to land mammals and birds. Both of them are animals. The exemplars of land mammals and birds have many similar surface features in perception. They have similar shapes. They both have heads, body and legs. But from the animal classification perspective, they belong to two different categories. So we predicted that there was also a tracking facilitation effect with this categorical difference.

### Participants

Eleven undergraduate students (five female) that did not participate in Experiments 1 and 2 aged 18–24 years (mean age = 22.27 years, *SD* = 1.79) with normal or corrected-to-normal vision completed the experiment. All observers provided informed written consent. The study was approved by the Institutional Review Board (Ethics Committee) of the School of Psychology at Beijing Normal University. All observers received payment.

### Design, Equipment, Stimuli, and Procedures

In Experiment 3, the objects were land mammals and birds. The eight land mammals were the same as those used in Experiments 1 and 2. The eight bird pictures were of a bird (a generic flying bird), chicken, duck, eagle, ostrich, owl, swan, and penguin, selected from Cycowicz et al.’s (1997) set of 400 line drawings. The design, equipment and procedures of Experiment 3 were the same as those in Experiments 1 and 2 (see **Table 5**).

**Table 5:** The eight conditions of Experiment 3.

Conditions	Levels	Targets	Distractors
Intra-category	Homogenous	Land mammals A, land mammals A, land mammals A, land mammals A	Land mammals B, land mammals B, land mammals B, land mammals B
	Four-unique	Land mammals A, land mammals A, land mammals B, land mammals B	Land mammals C, land mammals C, land mammals D, land mammals D
	All-unique	Land mammals A, land mammals B, land mammals C, land mammals D	Land mammals E, land mammals F, land mammals G, land mammals H
	Paired-two	Land mammals A, land mammals A, land mammals B, land mammals B	Land mammals A, land mammals A, land mammals B, land mammals B
Inter-category	Homogenous	Land mammals A, land mammals A, land mammals A, land mammals A	Bird A, bird A, bird A, bird A
	Four-unique	Land mammals A, land mammals A, land mammals B, land mammals B	Bird A, bird A, bird B, bird B
	All-unique	Land mammals A, land mammals B, land mammals C, land mammals D	Bird A, bird B, bird C, bird D
Homogenous		Land mammals A, land mammals A, land mammals A, land mammals A	Land mammals A, land mammals A, land mammals A, land mammals A


*The land mammals and birds targets and distractors were randomly assigned, although here only one particular combination is shown. Please see the text for more details.*

### Results and Discussion

**FIGURE 3:**
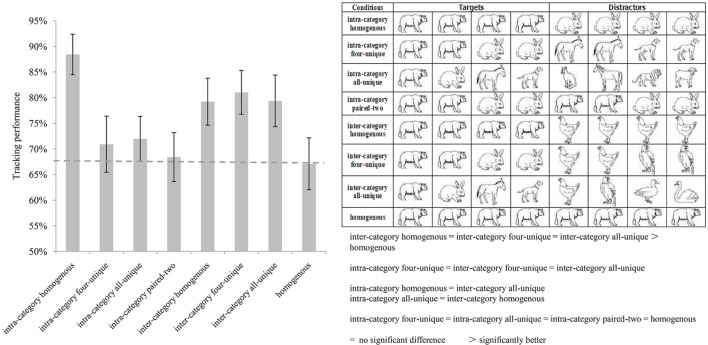
**The results of Experiment 3.** The **(left)** histogram describes the average tracking performance of each condition. The **(right)** above table illustrates the targets and distractors’ identities of each condition. The **(right)** below is the brief results of pairwise comparisons.

The results of Experiment 3 are presented in **Figure 3**. Similar to the results of Experiments 1 and 2, a repeated measures ANOVA indicated a significant main effect [*F*(7,70) = 22.499, *p* < 0.001, η^2^ = 0.692]. *Post hoc* tests with Bonferroni correction (see **Table 6**) also showed that tracking performances of the inter-category homogenous, inter-category four-unique, and inter-category all-unique conditions had no pairwise significant differences, *ps* > 0.05. The tracking performances of the intra-category four-unique, intra-category all-unique, intra-category paired-two, and homogenous conditions all had no pairwise significant differences, *ps* > 0.05. The tracking performances of the intra-category four-unique, the inter-category four-unique, and the inter-category all-unique also had no pairwise significant differences, *ps* > 0.05.

**Table 6:** The pairwise comparisons of conditions in Experiment 3.

	Intra-category homogenous	Intra-category four-unique	Intra-category all-unique	Intra-category paired-two	Inter-category homogenous	Inter-category Four-unique	Inter-category all-unique
Intra-category homogenous							
Intra-category four-unique	0.175^∗∗∗^						
Intra-category all-unique	0.165^∗∗∗^	-0.010					
Intra-category paired-two	0.200^∗∗∗^	0.025	0.035				
Inter-category homogenous	0.092^∗∗^	-0.083^∗^	-0.073	-0.108^∗^			
Inter-category four-unique	0.074^∗^	-0.101	-0.091^∗^	-0.126^∗^	-0.018		
Inter-category all-unique	0.091	-0.084	-0.074^∗^	-0.109^∗^	-0.001	0.017	
Homogenous	0.213^∗∗∗^	0.038	0.048	0.013	0.121^∗^	0.139^∗^	0.123^∗^


*The values in the table are the *mean differences* of tracking performances between two conditions. ^∗^*p* < 0.05, ^∗∗^*p* < 0.01, ^∗∗∗^*p* < 0.001.*

Moreover, no significant differences were found between the intra-category homogenous vs. inter-category all-unique conditions and the intra-category all-unique vs. inter-category homogenous conditions.

The tracking performances in the inter-category conditions were significantly better than that of the homogenous condition, suggesting that the categorical distinction between the targets and distractors enhanced tracking performance, even when the categorical difference was between two kinds of animals: land mammals and birds. However, there are some exceptions, such as no significant improvement was found between the intra-category four-unique vs. inter-category four-unique conditions and the intra-category four-unique vs. inter-category all-unique conditions; and accordingly, the category-based tracking facilitation effect decreased to some extent, compared to Experiments 1 and 2. One possible explanation is that the different animal exemplars had a similar overall shape and more detailed category which we seldom categorized them accurately in daily life according to observers’ general knowledge, then rendering identity differentiation difficulty.

## Experiment 4

Since the typical and nameable cartoon pictures were used as tracking objects in the present study, both the visual distinctiveness and semantic categories between targets and distractors in the inter-category conditions were larger than that of intra-category and homogenous conditions, the tracking benefits of inter-category conditions might be caused by visual distinctiveness among the pictures. To distinguish between the effects of categorical distinction and visual distinctiveness, we inverted the objects (targets and distractors) in Experiment 4 and compared the tracking facilitation effects of upright and inverted objects. The inverted pictures weaken the category representation but leave all the low-level features and consequently the visual distinctiveness intact. If the objects’ perceptual or visual distinctiveness was the only source of the tracking benefit, the inter-category tracking facilitation effect of upright and inverted objects should have no difference. However, if the upright inter-category facilitation effect was significantly larger than the inverted one, further semantic processes might be involved in the task and affected observers’ tracking performances.

### Participants

Twelve undergraduate students (seven female) aged 18–25 years (mean age = 22.54 years, *SD* = 1.83) with normal or corrected-to-normal vision completed the experiment. They all did not participate in Experiments 1, 2, and 3. All observers provided informed written consent. The study was approved by the Institutional Review Board (Ethics Committee) of the School of Psychology at Beijing Normal University.

### Design, Equipment, Stimuli, and Procedures

Three upright conditions were included in Experiment 4: intra-category all-unique condition where eight unique objects were from the same category, inter-category all-unique condition where the targets were four unique land mammals/furniture images and the distractors were four unique furniture/land mammals images, and homogenous condition where all of the objects were the same. The homogenous condition provided the performance baseline for comparison. These three conditions were the same as those in Experiment 1. In addition to the upright conditions, three corresponding inverted conditions were also examined. Both targets and distractors were upside down in the inverted conditions. Overall, the equipment, stimuli and procedures utilized in Experiment 4 were identical to those of Experiment 1.

The upright and inverted conditions were run in two blocks of trials. Observers always completed the inverted block first and then the upright block. Each block began with 6 practice trials and was followed by 60 randomly displayed experimental trials (20 trials × 3 levels of semantic category distinction).

### Results and Discussion

**FIGURE 4:**
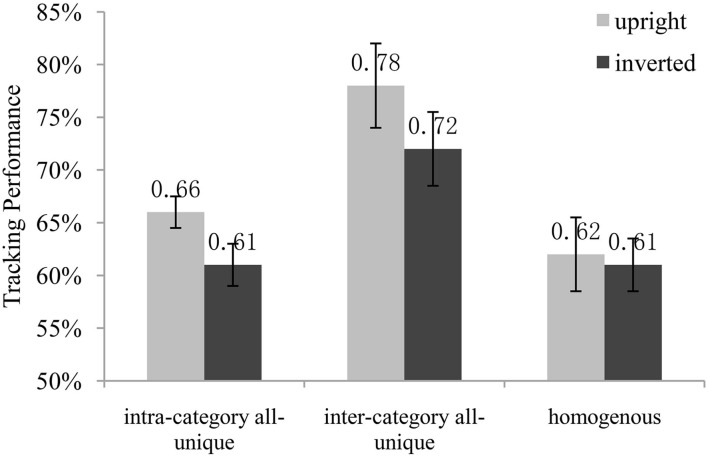
**The results of Experiment 4**.

The results of Experiment 4 are presented in **Figure 4**. A two-way repeated-measures ANOVA revealed a significant main effect of the upright/inverted factor, *F*(1,11) = 18.580, *p* = 0.001, η^2^ = 0.628, suggesting that observers’ tracking performances were better in the upright conditions than the inverted conditions. The main effect of the categorical distinction factor was also significant, *F*(2,22) = 36.796, *p* < 0.001, η^2^ = 0.770, and so was the interaction between the two factors, *F*(2,22) = 4.072, *p* = 0.031, η^2^ = 0.270.

*Post hoc* multiple comparisons (Bonferroni correction) revealed that, in the inverted conditions, the tracking performance of inter-category all unique was significantly better than that of intra-category (*MD* = 0.106, *p* < 0.001) and homogenous (*MD* = 0.112, *p* = 0.002). There was no significant difference between intra-category and homogenous (*MD* = 0.006, *p* = 1.000). The results of the upright conditions were consistent with Experiment 1. Tracking performance of inter-category was significantly better than that of intra-category (*MD* = 0.125, *p* < 0.001) and homogenous (*MD* = 0.165, *p* < 0.001), and there was also no significant difference between intra-category and homogenous (*MD* = 0.040, *p* = 0.209).

Simple main effects analyses revealed that the tracking performances between the inverted and upright homogenous conditions had no significant difference [*F*(1,11) = 0.22, *p* = 0.652]. However, for both intra-category and inter-category conditions, the tracking performances of observers with the upright objects were significantly better than that with the inverted objects (upright and inverted intra-category: *F*(1,11) = 24.52, *p* < 0.001; upright and inverted inter-category: *F*(1,11) = 23.76, *p* < 0.001). If only the perceptual process was involved in the tracking task, the tracking performances of upright and inverted conditions should have no difference. So these results suggested that the cognitive processing of objects with semantic meaning entered into the semantic level, and not just stayed on the perceptual level.

To further compare the difference of inter-category facilitation effect between the upright and inverted conditions, we introduced the performance difference between inter-category and homogenous conditions (inter-category minus homogenous) as the index of inter-category facilitation effect. *T*-test revealed that the upright inter-category facilitation effect (*MD* = 0.165, upright inter-category minus upright homogenous) was significantly larger than the inverted condition (*MD* = 0.112, inverted inter-category minus inverted homogenous) [*t*(11) = 2.788, *p* = 0.018]. Since the visual distinctiveness and perceptual process were the same for the upright and inverted objects, If only the perceptual difference was involved in the tracking task, the upright and inverted inter-category facilitation effects should have no difference. So we conjecture that the stronger upright inter-category facilitation effect was caused by the categorical distinction between the targets and distractors. The above two results provided evidences that besides the visual distinctiveness, the semantic category processing was also involved in the task and contributed the tracking benefit in the inter-category conditions.

## General Discussion and Conclusion

The goal of this study was to examine whether tracking performance would be enhanced when there were categorical or non-categorical distinctions between the targets and distractors. Experiment 1 established the principal finding that categorical distinction between targets and distractors significantly facilitated observers’ tracking performance and that a category-based grouping effect might exist during tracking. In Experiment 2, a tracking facilitation effect was observed in the inter-category conditions when the categorical difference between the targets and distractors narrowed from natural and artificial objects to land mammals and fruits. The results of Experiment 3 were consistent with those of Experiments 1 and 2, except that the tracking performance in the intra-category homogenous condition (the targets were four same land mammals or birds, while the distractors were another four same land mammals or birds in the same category) was significantly better than that in the inter-category conditions, when the categorical difference was further narrowed to land mammals and birds, both of which are animals. That is, the identity difference of the targets and distractors played a more important role in facilitating tracking than categorical information. Thus, the categorical distinction advantage narrowed with the decreasing categorical difference.

The results of Experiment 4 provided evidence that tracking facilitation of the targets and distractors chosen from different categories was not only caused by the visual distinctiveness. Although tracking facilitation effect existed in both upright and inverted conditions, observers displayed a significantly larger inter-category facilitation effect in the upright condition than in the inverted condition. This result suggested that, besides the perceptual grouping process, objects’ semantic category information also play an important role during tracking. It also indicated that the cognitive processing of objects in dynamic scenes is not just staying on the perceptual level, but enter to a higher level of object recognition and semantic classification.

The present results supported our category-based grouping hypothesis. It was demonstrated that the human visual system improved tracking performance as semantic category difference between targets and distractors increased. This tracking facilitation effect was detected even when the categorical difference was relatively small. We propose that the tracking facilitation effect in the inter-category conditions compared to the intra-category conditions (according to the results of Experiments 1, 2, and partial results from Experiment 3) was due to the processing of the objects’ semantic category. The reasoning will be illustrated in the following paragraphs. The grouping hypothesis proposed by Yantis (1992) highlights the importance of perceptual grouping, which allows the targets to be treated as a unit by the visual system. In addition to the original spatiotemporal information, surface feature information, such as color, size, shape, and interpolation, can also automatically bind targets and distractors into one group (Keane et al., 2011; Erlikhman et al., 2013). However, in Keane et al. (2011) and Erlikhman et al. (2013), the grouping effect was not only based on the different features between the targets and distractors, but also on the spatiotemporal properties. For example, in a study by Erlikhman et al. (2013), displays contained four pairs of objects, one in each screen quadrant, and the objects orbited around a central point in each quadrant during the motion phase. Spatiotemporal properties could facilitate grouping compared to randomly moving trajectories.

In the current study, all of the objects moved randomly and independently, no spatiotemporal information could promote the perceptual grouping of targets. We manipulated the categorical differences systemically from animals vs. furniture, animals vs. fruits to land mammals vs. birds and found the consistent inter-category facilitation effect, even when the features of two categories were similar in perception (as shown in Experiment 3). Therefore, it is very likely that observers’ processing of the objects’ semantic category causes the facilitation effect detected in Experiments 1–3. Specifically, based on our results, we conjecture that there exists a category-based grouping mechanism during tracking when targets and distractors are categorical distinct. Observers used the categorical difference between the targets and distractors to organize them into two separate groups. They might treat the targets in the same category as one unit during tracking; therefore make the working memory load of targets lighter and the target recovery strategy easier and more effective (Makovski and Jiang, 2009a,b; Ren et al., 2009; Liu et al., 2012). Once one or more targets were lost, observers could find them according to their semantic category. In our category-based grouping hypothesis, the grouping process was accompanying by and interacting with working memory mechanism. They are not two separate or conflicting processes. The grouping of targets could improve the memory for the moving objects; therefore make the lost targets be found much accurately and resulted a higher tracking performance. However, further studies are needed to examine the mechanism of the tracking facilitation effect.

The results of Experiments 1, 2, and 3 also consistently showed that except the tracking performance of intra-category homogenous condition significantly better than the homogenous condition, there was no significant difference among other intra-category conditions and the homogenous condition. This result was inconsistent with a previous finding that unique objects could aid tracking in MIT tasks (Horowitz et al., 2007; Drew et al., 2013), and might reflect the limitations of our study. Specifically, there might be two reasons for this inconsistency. One is that observers were not required to report the identities of the targets after tracking in the current task. That is, they need not to bind the targets’ identities and locations together. Previous researches suggest that two separate systems might be employed during identity tracking: the tracking of targets’ location and identity processing (Horowitz et al., 2007; Cohen et al., 2011). Therefore, identity processing during tracking is involuntary to some extent. Whether it could aid tracking or not, the identities of the targets are processed, even when the processing of identities requires additional resources and impairs tracking performance (Ren et al., 2009; Liu et al., 2012). However, in the present study, when the identities of the targets were not required to report, they might not be fully processed and utilized. The second reason is that the size of the objects used in our experiment was relatively small (2.4° × 2.4°). Moreover, the objects’ speed of motion was high. On this occasion, the effect of the objects’ identities on tracking performance might decrease. Even though after the experiments, all observers orally reported that they could discriminate the objects clearly during tracking, this finding reflects two limitations of the present study.

Multiple Identity Tracking study in semantic and conceptual level is a new idea in the realm of cognitive processing of visual objects. This topic is worthy to investigate from both the theoretical and practical perspectives. The results of the present study suggested that observers could use the semantic categorical difference between the targets and distractors to improve their tracking performance in MIT tasks. This tracking facilitation effect decreased when the categorical distinction was narrowed. Observers might organize the targets of the same category into one group and treat them as a whole during tracking. They might find the lost objects more quickly and accurately based on their conceptual category.

## Author Contributions

Conceived and designed the experiments: LW, XZ. Performed the experiments: LW, CL. Analyzed the data: LW. Contributed to the writing of the manuscript: LW, XZ, CL, ZL.

## Conflict of Interest Statement

The authors declare that the research was conducted in the absence of any commercial or financial relationships that could be construed as a potential conflict of interest.
